# PIN1 transcript variant 2 acts as a long non-coding RNA that controls the HIF-1-driven hypoxic response

**DOI:** 10.1038/s41598-019-47071-1

**Published:** 2019-07-22

**Authors:** Yong-Joon Choi, Iljin Kim, Jae Eun Lee, Jong-Wan Park

**Affiliations:** 1 Department of Biomedical Sciences, Seoul National University College of Medicine, Seoul, Republic of Korea; 2 Department of Pharmacology, Seoul National University College of Medicine, Seoul, Republic of Korea; 3 Cancer Research Institute and Ischemic/Hypoxic Disease Institute, Seoul National University College of Medicine, Seoul, Republic of Korea; 4 BK21-plus Biomedical Science Project, Seoul National University College of Medicine, Seoul, Republic of Korea

**Keywords:** Mechanisms of disease, Long non-coding RNAs

## Abstract

The transcription factor HIF-1 induces the expression of genes that are essential for cell survival and oxygen homeostasis in hypoxic conditions. The prolyl isomerase Pin1 plays a role in the regulation of HIF-1α. However, the mechanism by which Pin1 controls HIF-1α remains controversial. Surprisingly, we here show that a *PIN1* transcript downregulates HIF-1α as a long non-coding RNA. Pin1-silencing siRNAs augmented the hypoxia-induced expression of HIF-1α, thereby upregulating the expression of HIF-1 target genes. However, the overexpression of Pin1 protein did not inhibit the hypoxic expression of HIF-1α. Pin1 restoration in Pin1-depleted cells also failed to reverse the induction of HIF-1α by Pin1 knockdown. Unexpectedly, HIF-1α was found to be induced by both siRNAs for *PIN1* transcript variants 1/2 and that for *PIN1* transcript variants 2/3, indicating that the *PIN1* transcript variant 2 (*PIN1*-v2) is responsible for HIF-1α induction. Mechanistically, *PIN1*-v2, which is classified as a long non-coding RNA due to early termination of translation, was evaluated to inhibit the transcription of *HIF1A* gene. In conclusion, *PIN1*-v2 may function in balancing the HIF-1-driven gene expression under hypoxia.

## Introduction

Hypoxia-inducible factor-1 (HIF-1) is a heterodimeric transcription factor that responds to cellular oxygen concentrations^1^. HIF-1 is composed of the oxygen-regulated α subunit (HIF-1α) and the constitutively expressed β subunit (HIF-1β). In aerobic conditions, HIF-1α is hydroxylated at prolines 402 and 564 by oxygen-sensitive prolyl hydroxylase (PHD) enzymes, poly-ubiquitinated by an E3 ligase von Hippel-Lindau protein (pVHL), and degraded by the 26S proteasome^2,3^. Under hypoxia, however, the PHD-mediated hydroxylation is blocked due to lack of the substrate oxygen. Consequently, HIF-1α escapes the ubiquitin-proteasome system (UPS). The stabilized HIF-1α enters into the nucleus and associates with HIF-1β to form the transcription factor HIF-1. Hundreds of HIF-1 target genes have been identified to be activated upon hypoxic stress to regulate various biological processes such as angiogenesis, energy metabolism, and cell proliferation^4^.

Peptidyl-prolyl cis-trans isomerase NIMA-interacting 1 (Pin1) is an enzyme that possesses an N-terminal WW domain and a C-terminal isomerase domain. The 39-residue WW domain contains two conserved tryptophan (W) residues spaced 22 amino acids apart. This domain functions as a protein interaction platform that recognizes a phosphoserine/phosphothreonine-proline (pSer/pThr-Pro) motif within the target proteins. The isomerase domain catalyzes the cis-trans conversion of the proline residue in the pSer/pThr-Pro motif of the target protein^5^. The phosphorylation of Ser/Thr-Pro motif, which is mediated by a variety of Ser/Thr-Pro kinases such as cyclin-dependent protein kinases (CDKs), extracellular signal-regulated kinases (ERKs), glycogen synthase kinases (GSKs), and Polo-like kinases (PLKs), is an essential prerequisite for the prolyl cis-trans conversion. This cis-trans conversion makes a significant change in protein conformation and affects protein stability or function^6^. Because HIF-1α is phosphorylated at Ser/Thr-Pro motifs by GSK-3β or MAPK^7^, recent studies have demonstrated that Pin1 regulates HIF-1a by altering its structure^8–10^. Initially, Lonati *et al*. reported that Pin1 physically interacts with HIF-1α in primary rat hippocampal cells and thereby destabilizes the protein^8^. In contrast, Jalouli *et al*. reported that Pin1 enhances the transcriptional activity of HIF-1α without affecting HIF-1α expression in HeLa cells^9^. In another report, Pin1 was shown to stabilize HIF-1α and subsequently enhance the transcription of HIF-1 target genes in HCT116 cells^10^. Therefore, the role of Pin1 in the HIF-1 signaling pathway still remains unclear.

To clarify the mechanism by which Pin1 regulates HIF-1-driven adaptation to hypoxia, we performed a molecular work in the present study, and found an unexpected role of Pin1 in HIF-1α regulation. Of the three splicing variants of *PIN1* gene, the variant 2 was identified as a long non-coding RNA that downregulates HIF-1α under hypoxia. These results may have further implications for the treatment of hypoxia-related diseases.

## Results

### Pin1-targeting siRNAs increase HIF-1α protein level and transcriptional activity under hypoxia

**Figure 1:**
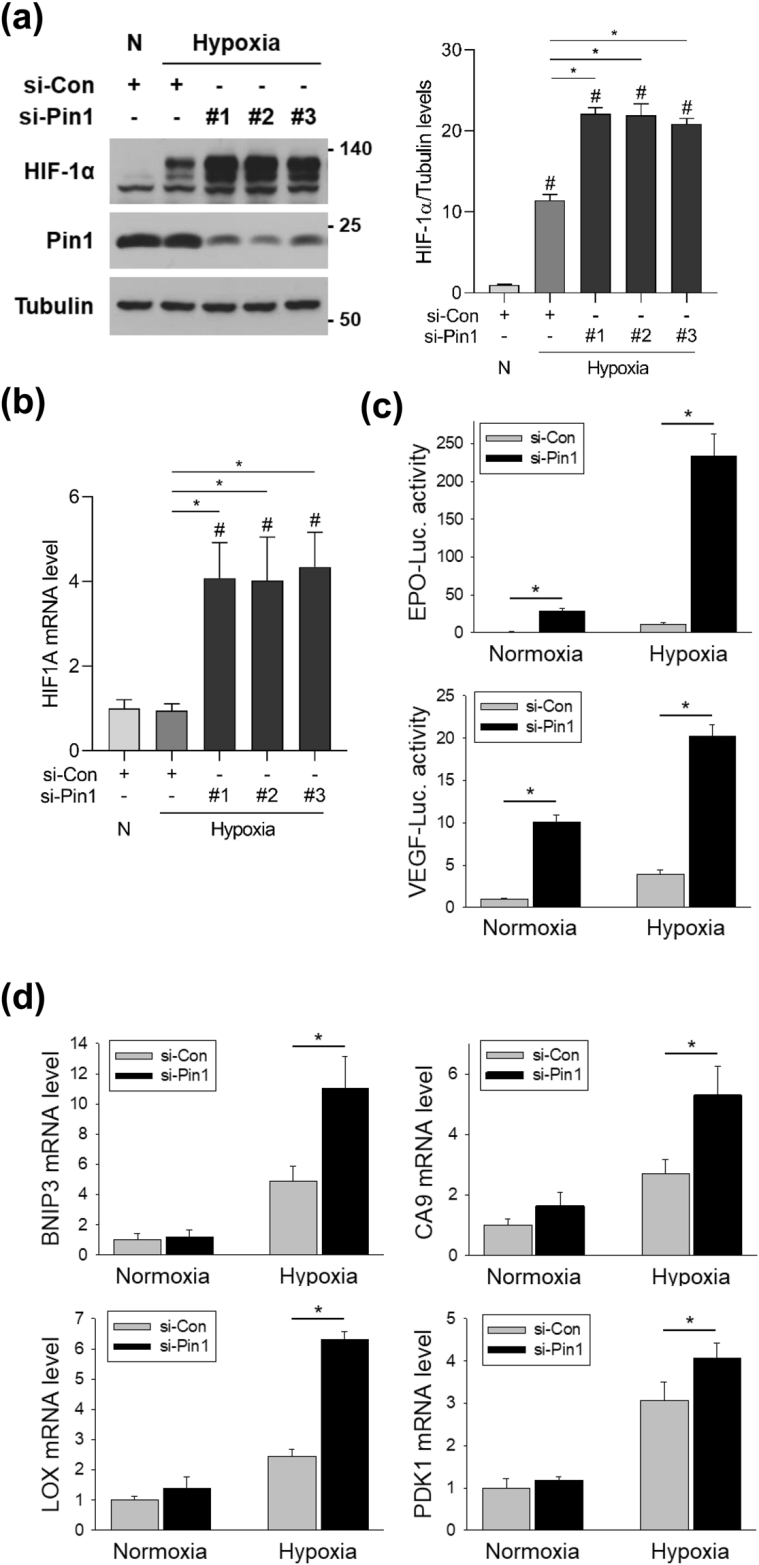
Pin1 knockdown upregulates HIF-1α and facilitates HIF-1-driven gene expression. (**a**) HEK293 cells were transfected with the indicated siRNAs, incubated in normoxia or hypoxia (1% O_2_) for 8 h, and subjected to western blotting. HIF-1α blots were quantified using ImageJ and displayed in bar graph (mean ± SD, n = 3). ^#^*P* < 0.05 compared to the control group; *P < 0.05 by Student’s t-test. (**b**) Cells were treated as in (a) and *HIF1A* mRNA expression was measured by RT-qPCR. Data represent mean ± SD (n = 3). ^#^*P* < 0.05 compared to the control group; *P < 0.05 by Student’s t-test. (**c**) Cells were transfected with the *EPO* enhancer (or *VEGF* promoter)-luciferase plasmid, the CMV-β-galactosidase plasmid, and the indicated siRNAs. After cells were incubated in normoxia or hypoxia for 16 h, luciferase activities were measured, and normalized to β-galactosidase activities. Data represent mean ± SD (n = 3). *P < 0.05 by Student’s t-test. (**d**) Cells were transfected with the indicated siRNAs, incubated in normoxia or hypoxia for 16 h, and lysed for RNA extraction. *BNIP3*, *CA9*, *LOX*, and *PDK1* mRNA expression levels were measured by RT-qPCR. Data represent mean ± SD (n = 3). *P < 0.05 by Student’s t-test.

To investigate the role of Pin1 in HIF-1α regulation, we first tested the effect of siRNA-mediated Pin1 knockdown. HEK293 cells were transfected with three different siRNAs targeting Pin1. The siRNAs, all of which efficiently decreased Pin1 expression, increased the protein level (Fig. 1a) and mRNA expression (Fig. 1b) of HIF-1α under hypoxia. To evaluate the functionality of HIF-1α, we used luciferase reporter plasmids harboring a hypoxia response element (HRE) originating either from the erythropoietin (*EPO*) enhancer or the vascular endothelial growth factor (*VEGF*) promoter. EPO is a hormone secreted by the kidney in response to hypoxiathat induces the production of red blood cells. VEGF, also induced by hypoxia, is a signaling protein that stimulates the formation and growth of blood vessels. In hypoxia, HIF-1 binds to the HRE of these genes and upregulates their expression. Both HIF-1 reporters were activated by Pin1 knockdown in both normoxia and hypoxia (Fig. 1c). Furthermore, Pin1 knockdown augmented the hypoxic induction of HIF-1 target genes, such as *BNIP3* (BCL2 Interacting Protein 3), *CA9* (Carbonic Anhydrase 9), *LOX* (Lysyl Oxidase), and *PDK1* (Pyruvate Dehydrogenase Kinase 1) (Fig. 1d). These data strongly indicate that Pin1 negatively regulates HIF-1α expression and HIF-1 target gene expression.

### Pin1 overexpression does not affect HIF-1α expression

**Figure 2:**
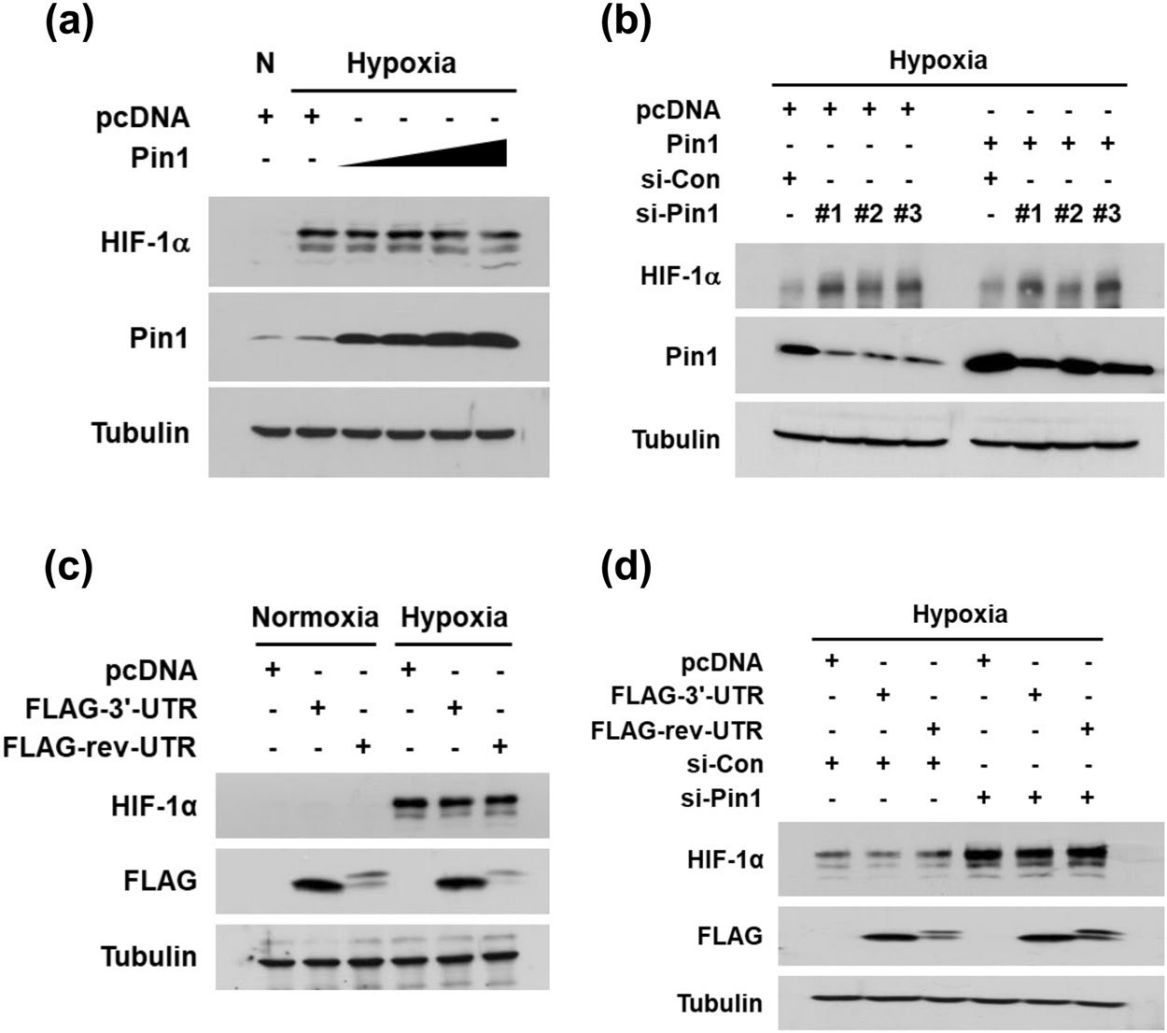
Pin1 overexpression does not affect HIF-1α expression. (**a**) HEK293 cells were transfected with Pin1 plasmids (0.2, 0.5, 1, 2 μg per 100 mm dish), incubated in hypoxia for 8 h, and subjected to western blotting. (**b**) Cells were transfected with the indicated plasmids and siRNAs, incubated in hypoxia for 8 h, and subjected to western blotting. (**c**) Cells were transfected with the FLAG-fused 3′-UTR of *PIN1* variant 1 or the FLAG-fused reverse sequence of the 3′-UTR plasmid. After cells were incubated in normoxia or hypoxia for 8 h, proteins were analyzed by western blotting. (**d**) Cells were transfected as indicated, incubated in hypoxia for 8 h, and subjected to western blotting. The expression of *PIN1* 3′-UTR was verified by examining FLAG expression.

As HIF-1α was upregulated by Pin1 knockdown, it was expected that HIF-1α would be downregulated by Pin1 overexpression. Strangely, however, the transfection with the Pin1 plasmid failed to reduce the level of HIF-1α protein in HEK293 cells (Fig. 2a). To examine whether Pin1 overexpression reverses the induction of HIF-1α by Pin1 knockdown, we co-transfected HEK293 cells with Pin1 siRNA and Pin1 plasmid. Although Pin1 expression fully recovered after the transfection, the effects of Pin1 siRNAs on HIF-1α expression were not abolished (Fig. 2b). This suggests that Pin1 protein is not involved in HIF-1α regulation, so we considered the possibility that the transcripts of the *PIN1* gene regulate HIF-1α expression. We checked if the 3′ untranslated region (3′-UTR) of the *PIN1* transcript regulates HIF-1α expression. However, the expression of the *PIN1* 3′-UTR and its complementary RNA (rev-UTR) did not affect the HIF-1α levels in both normal and Pin1-depleted conditions (Fig. 2c,d).

### Knockdown of *PIN1* transcript variant 2 upregulates HIF-1α and its downstream genes

**Figure 3:**
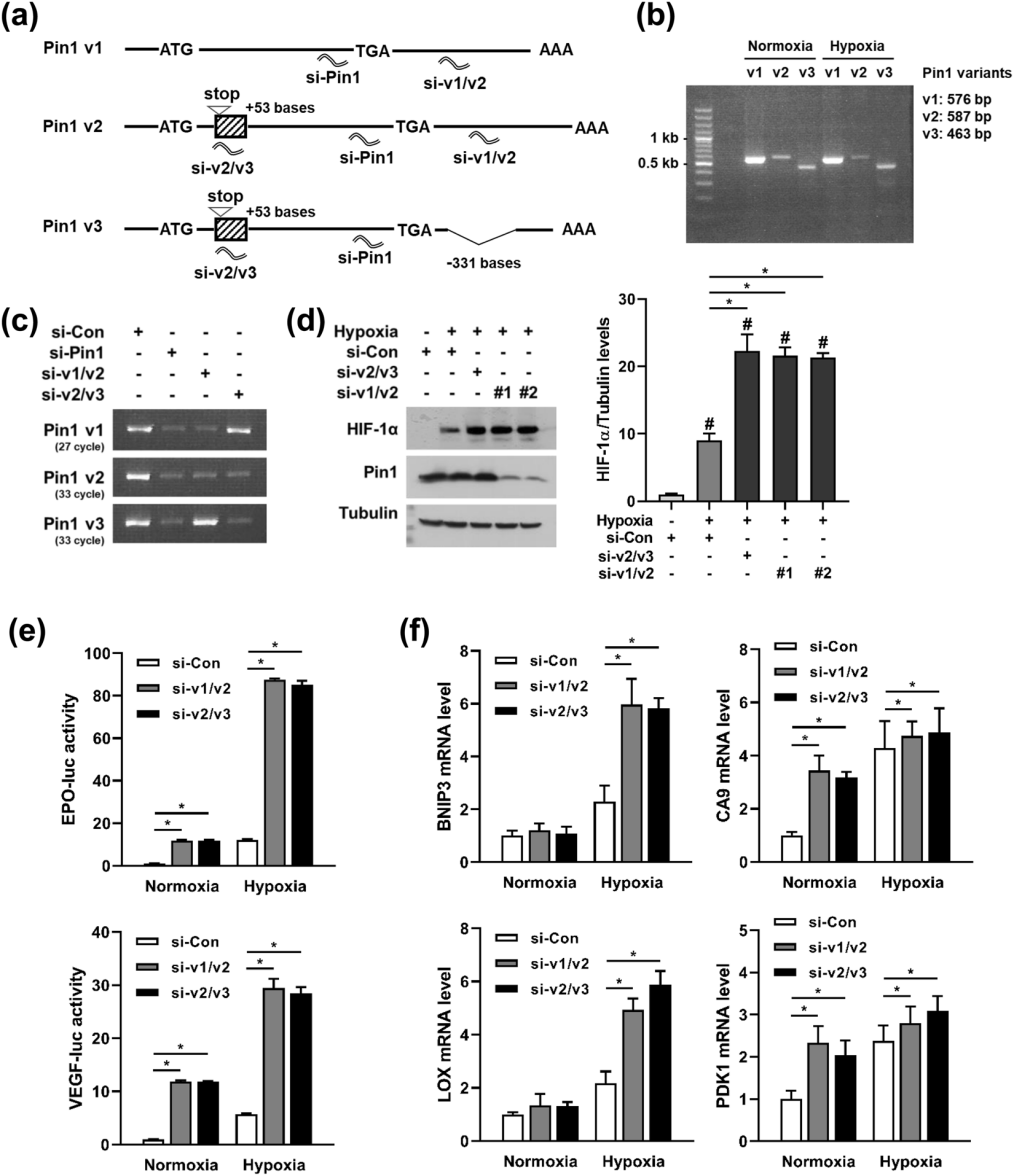
The knockdown of *PIN1*-v2 upregulates HIF-1α and increases the HIF-1-driven gene expression. (**a**) Schematic illustration of the splicing variants encoded by the *PIN1* gene. The striped boxes indicate the additional segment included in *PIN1*-v2/v3, which contains the stop codon. Si-Pin1 is the siRNA for all the variants, si-v2/v3 specifically for *PIN1*-v2/v3, and si-v1/v2 specifically for *PIN1*-v1/v2. (**b**) Transcript variants of the *PIN1* gene in HEK293 cells were amplified by RT-PCR and the PCR products were electrophoresed and stained with ethidium bromide. (**c**) HEK293 cells were transfected with the indicated siRNAs and lysed for RNA extraction. Knockdown of *PIN1* variants were verified by RT-PCR and electrophoresis. (**d**) Cells were transfected with the indicated siRNAs, incubated in normoxia or hypoxia for 8 h, and subjected to western blotting. Immunoblots were quantified using ImageJ and displayed in bar graph (mean ± SD, n = 3). ^#^*P* < 0.05 compared to the control group; *P < 0.05 by Student’s t-test. (**e**) Cells were transfected with the reporter plasmids and the indicated siRNAs, incubated in normoxia or hypoxia for 16 h, and subjected to luciferase assay. The normalized luciferase activities are presented in bar graphs (mean ± SD, n = 3). *P < 0.05 by Student’s t-test. (**f**) Cells were transfected with the indicated siRNAs, incubated in normoxia or hypoxia for 16 h and lysed for RNA extraction. *BNIP3*, *CA9*, *LOX*, and *PDK1* mRNA expression levels were measured by RT-qPCR. Data represent mean ± SD (n = 3). *P < 0.05 by Student’s t-test.

Of the three transcript variants in *PIN1*, the variant 1 (*PIN1*-v1) encodes the wild-type Pin1 protein, but the variants 2 (*PIN1*-v2) and 3 (*PIN1*-v3) do not generate functional proteins due to early termination of translation. Therefore, *PIN1*-v2 and v3 are regarded as long non-coding RNAs (lncRNAs). As shown in Fig. 3a, *PIN1*-v2 and v3 contain an extra sequence, which includes a premature translation-stop codon, following the translation-start codon. In addition, *PIN1*-v3 lacks a segment (331 bases) at the 3′-UTR region (Supplementary Fig. S1). We performed RT-PCR to verify the presence of these *PIN1* variants in cells (Fig. 3b). In order to investigate the potential function of *PIN1* variants in HIF-1α regulation, we designed an siRNA specifically targeting *PIN1*-v2 and v3 (hereafter abbreviated as ‘si-v2/v3′). The specificity of the siRNA was verified by RT-PCR and electrophoresis (Fig. 3c). Interestingly, despite the unchanged expression of Pin1 protein, si-v2/v3 upregulated HIF-1α under hypoxia (Fig. 3d). We confirmed the effect of si-v2/v3 in eight additional cell lines (Supplementary Fig. S2). Next, we designed another siRNA (abbreviated as ‘si-v1/v2’) targeting *PIN1*-v1 and *PIN1*-v2, but not *PIN1*-v3, to identify which of two variants takes part in HIF-1α regulation (Fig. 3a,c). Unfortunately, it is impossible to design an siRNA that targets only one of the three variants. Nevertheless, we found that both si-v1/v2 and si-v2/v3 increased the protein levels of HIF-1α (Fig. 3d). Because the two siRNAs commonly silence *PIN1*-v2, *PIN1*-v2 is likely to be responsible for HIF-1α induction. As per the luciferase reporter assays, the transcriptional activity of HIF-1α was increased in HEK293 cells transfected with either si-v1/v2 or si-v2/v3 (Fig. 3e). Further, the mRNA levels of HIF-1 target genes were increased by both siRNAs (Fig. 3f). The reporter and RT-qPCR assays were also conducted in DU145 cells, which showed similar results (Supplementary Fig. S3). Taken together, *PIN1*-v2 seems to act as a lncRNA to control HIF-1-driven adaptation to hypoxia.

### *PIN1* variant represses HIF-1α at the transcriptional level

**Figure 4:**
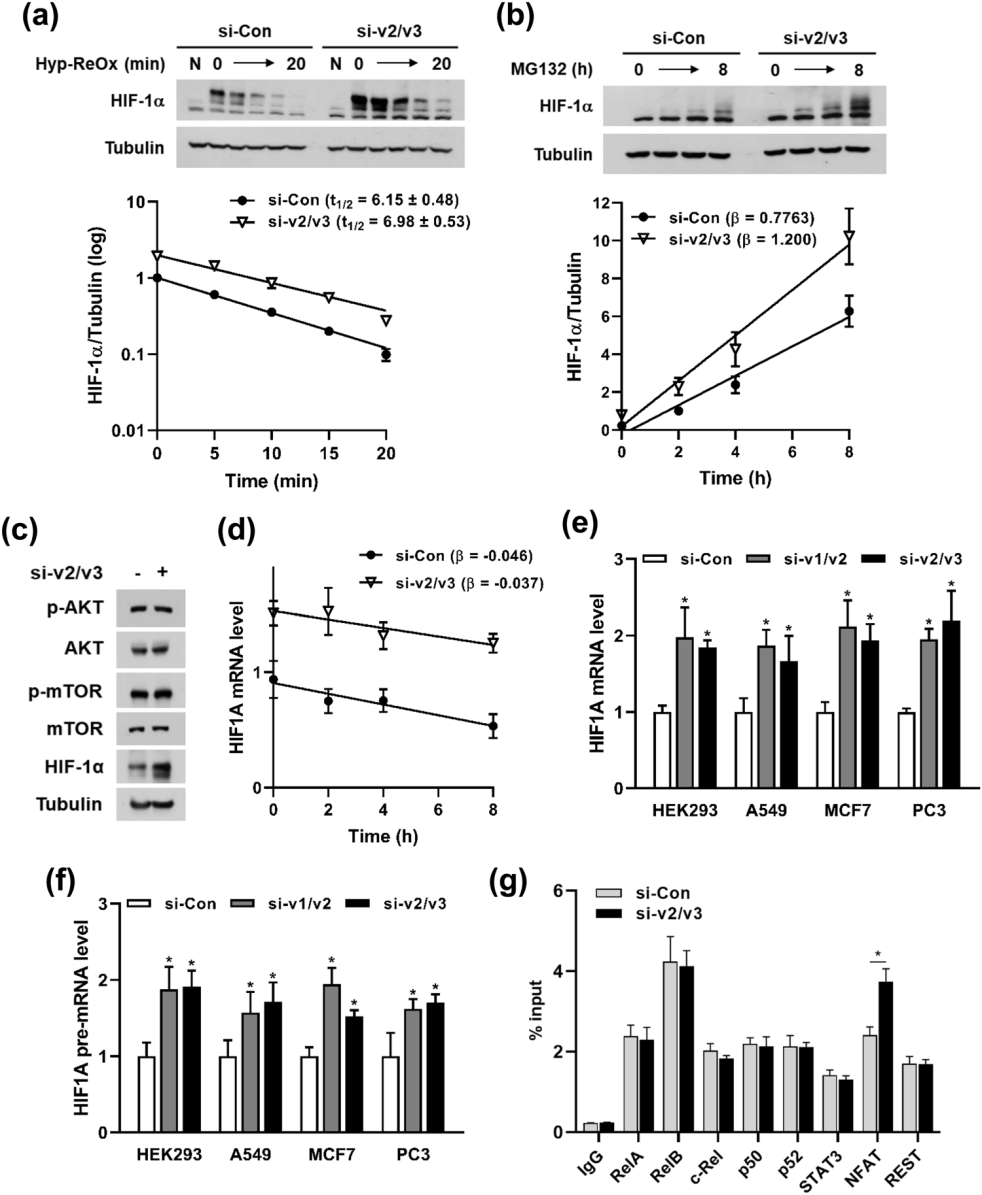
The knockdown of *PIN1* variant upregulates HIF-1α at the transcriptional level. (**a**) Degradation of hypoxia-stabilized HIF-1α protein was induced by reoxygenation. HEK293 cells were transfected with the indicated siRNAs and incubated in hypoxia for 8 h. While cells were re-oxygenated at 21% O_2_, cells were harvested at the indicated times and subjected to western blotting. HIF-1α protein levels were measured by checking blot intensity and plotted to the semi-log chart to linearize the first-order decay curve. Data represent mean ± SD (n = 3). The half-life t_1/2_ (mean ± SD) was calculated using GraphPad Prism. (**b**) Cells were transfected with the indicated siRNAs. After being treated with 10 μM MG132, cells were harvested at various periods (0, 2, 4, 8 h). HIF-1α/tubulin blot intensities (mean ± SD, n = 3) are shown in line graph at the lower panel with calculated slope values (β). (**c**) Cells were transfected with control or *PIN1*-v2/v3 siRNAs, and the levels of phosphorylated AKT and mTOR were analyzed by western blotting. (**d**) Decay rate of *HIF1A* mRNA was analyzed. Cells were transfected with indicated siRNAs and incubated with 5 μg/mL of actinomycin D for various periods (0, 2, 4, 8 h). *HIF1A* mRNA expression was measured by RT-qPCR and shown in line graph with calculated slope values (β). Data represent mean ± SD (n = 3). (**e**) HEK293, A549, MCF7, and PC3 cells were transfected with the indicated siRNAs and lysed for RNA extraction. *HIF1A* mRNA expression was measured by RT-qPCR. Data represent mean ± SD (n = 3). *P < 0.05 by Student’s t-test. (**f**) Cells were treated as in (e) and *HIF1A* pre-mRNA expression was measured by RT-qPCR. Data represent mean ± SD (n = 3). *P < 0.05 by Student’s t-test. (**g**) Cells were transfected with the indicated siRNAs and subjected to chromatin immunoprecipitation assay using indicated antibodies. *HIF1A* promoter region was detected by qPCR. Data represent mean ± SD (n = 3). *P < 0.05 by Student’s t-test.

Next, we investigated the mechanism by which the *PIN1* lncRNA regulates HIF-1α expression. To check the oxygen-dependent degradation of HIF-1α, we incubated HEK293 cells under hypoxia, followed by re-oxygenation. While cells were re-oxygenated, HIF-1α was quickly degraded in both si-v2/v3 and si-control groups. As there were no significant differences in the half-life of HIF-1α protein between two groups, we concluded that the *PIN1* variant does not regulate the process for oxygen-sensitive HIF-1α degradation (Fig. 4a). Next, we measured the de novo synthesis of HIF-1α protein. HEK293 cells were treated with a proteasome inhibitor MG132 to accrue HIF-1α protein without degradation. As shown in Fig. 4b, the accumulation of HIF-1α protein was faster in si-v2/v3-treated cells than in control cells. Similar results were observed when cells were treated with bafilomycin A1 to block the autophagic degradation of HIF-1α (Supplementary Fig. S4)^11^. These data strongly indicate that the *PIN1* variant negatively regulates the de novo synthesis of HIF-1α. Since the AKT/mTOR pathway determines the translation of the *HIF1A* mRNA^12^, we evaluated the activation of AKT and mTOR by checking their phosphorylated forms. However, si-v2/v3 did not affect this pathway (Fig. 4c). Next, cells were treated with actinomycin D to inhibit the de novo synthesis of *HIF1A* mRNA and the decay rate was calculated by RT-qPCR. As shown in line graph, si-v2/v3 did not affect the decay rate of *HIF1A* (Fig. 4d). To investigate whether the *PIN1* variant regulates HIF-1α at the transcriptional level, we performed RT-qPCR in four different cell lines. The si-v1/v2 and si-v2/v3 successfully increased *HIF1A* expression in most cell lines tested (Fig. 4e). Moreover, *HIF1A* pre-mRNA expression was also increased in these cell lines (Fig. 4f). These results suggest that the *PIN1*-v2 controls the HIF-1α expression at the transcriptional level. Lastly, we used ChIP-qPCR assay to find the transcription factor responsible for the si-v2/v3-mediated upregulation of HIF-1α. ChIP assay was performed to explore the effect of si-v2/v3 on the binding between the *HIF1A* promoter and previously reported nuclear factors that target *HIF1A* for transactivation^13–16^. Among the known factors, NF-κB subunits, STAT3, NFAT, and REST proteins successfully bound to the promoter region of *HIF1A*. However, si-v2/v3 increased only the interaction between NFAT and the *HIF1A* promoter, which may have contributed to the enhanced transcription of *HIF1A* mRNA (Fig. 4g). Taken together with the results shown in Fig. 3, we concluded that *PIN1*-v2 acts as the lncRNA responsible for the inhibition of the *HIF1A* transcription.

## Discussion

In this study, we aimed to clarify the controversial role of Pin1 in the HIF-1-driven cell adaptation to hypoxia. Pin1 knockdown reinforced the hypoxic induction of HIF-1α and subsequently enhanced the HIF-1-driven gene expression. However, Pin1 overexpression failed to repress HIF-1α expression, which prompted us to hypothesize that the *PIN1* transcripts, rather than the protein, regulate HIF-1α. Indeed, the knockdown of *PIN1*-v2 upregulated HIF-1α at the transcriptional level and increased the expression of HIF-1 target genes. Taken together, we conclude that *PIN1*-v2 plays a role in balancing the HIF-1-driven adaptation to hypoxia.

Using specific siRNAs targeting the common sequences in *PIN1*-v2/v3 and in *PIN1*-v1/v2, we demonstrated that *PIN1*-v2 regulates HIF-1α expression. As *PIN1*-v2 is not translated into a protein due to early termination, the variant may function as a lncRNA that modulates *HIF1A* transcription. Although lncRNAs were previously considered as non-functional RNA junk^17^, numerous studies have uncovered the important roles of lncRNAs in various biological processes^18^. However, many previously identified lncRNAs remain uncharacterized. Likewise, the biological functions of *PIN1*-derived lncRNAs have not been investigated. Here, we discovered a new function of the *PIN1* lncRNA in the HIF-1-mediated cellular response to hypoxia.

Several microRNAs have been reported to directly or indirectly regulate HIF-1α at the post-transcriptional level^19^. For example, the miR-17-92 family of microRNA clusters targets *HIF1A* mRNA to block its translation^20^. Therefore, we initially speculated that *PIN1* lncRNA might directly target and destabilize the *HIF1A* mRNA as microRNAs do. However, this does not seem to be the case as we observed that the pre-mRNA expression of *HIF1A* was increased by *PIN1*-v2/v3 knockdown. In addition, the decay rate of *HIF1A* mRNA was unaffected by si-v2/v3. Hence, it is more likely that the *PIN1* lncRNAs regulate HIF-1α at the transcriptional level. The transcription of *HIF1A* gene is known to be modulated by number of factors such as Angiotensin II, LPS, cytokines, RAF, NF-κB, and Src^21^. Recently, it has become apparent that lncRNAs also act as transcriptional regulators. They are known to target different aspects of the transcription process through interaction with the the DNA duplex, DNA binding proteins, transcription factors, and histone modifiers^22^. Here, we showed that *HIF1A* transcription is regulated by *PIN1* lncRNA. We expect that *PIN1* lncRNA interrupts NFAT recruitment to the *HIF1A* promoter, possibly (1) by creating steric hindrance between the two molecules; (2) by blocking the nuclear translocation of NFAT; or (3) by reducing the mRNA expression or protein levels of NFAT. The precise mechanism by which *PIN1* lncRNA inhibits *HIF1A* transcription remains to be investigated.

Our findings are contradictory to the results of a previous study showing that Pin1 knockdown reduced HIF-1α expression in HCT116 cells^10^. For this reason, we examined the effect of Pin1 knockdown on HIF-1α expression in ten different cell lines and found that Pin1 knockdown augments the hypoxic induction of HIF-1α in all cell lines tested. Although we cannot explain such a discrepancy on the effect of Pin1 knockdown, it is possible that the *PIN1* transcript variants are differentially expressed in HCT116 or that *HIF1A* transcription is regulated depending on cell context.

Therapeutic siRNAs that induce HIF-1α is a possible option in the development of new drugs for hypoxia-associated diseases. Indeed, the siRNA-based gene therapy has attracted significant interest from pharmaceutical companies^23–25^. In the case of chronic kidney disease (CKD)-associated anemia, EPO analogues or erythropoiesis-stimulating agents (ESA) have been used to increase RBC production from the bone marrow. However, since these therapeutics induce major side-effects, including elevated risk of cardiovascular diseases, the reinforcement of endogenous EPO production has been suggested as an alternative approach for the treatment of CKD-associated anemia^26–28^. For this purpose, the siRNA-based induction of renal HIF-1α may be a promising approach for treating anemia in CKD patients. In the present study, we propose that the siRNA targeting *PIN1* lncRNAs is a new therapeutic option for the management of CKD-associated anemia or other hypoxia-associated diseases.

## Materials and Methods

### Reagents and antibodies

HEK293, MCF7, A549, PNT2, CCD-18Lu, HaCaT, and U251 human cell lines were obtained from the American Type Culture Collection (Manassas, VA, United States) and cultured as per ATCC instructions. PC3 and DU145 cell lines were purchased from the Korean Cell Line Bank (Seoul, South Korea). Human astrocytes were obtained from Applied Biological Materials (Richmond, Canada) and cultured in type I collagen-coated dishes (Millipore, Billerica, MA, United States). MG132 (C2211), bafilomycin A1 (B1793), and actinomycin D (A9415) were purchased from Sigma–Aldrich (St. Louis, MO, United States). The following antibodies were used: anti-Pin1 (sc-15340), anti-β-tubulin (sc-9104), anti-GFP (sc-9996), anti-RelA (sc-8008), anti-c-Rel (sc-6955), anti-p50 (sc-1190), anti-p52 (sc-7386), anti-NFAT (sc-7294), and anti-REST (sc-374611) from Santa Cruz Biotechnology (Dallas, TX, United States); anti-AKT (9272), anti-phospho-AKT (9271), anti-mTOR (2972), anti-phosphor-mTOR (2971), anti-RelB (10544), and anti-STAT3 (9139) from Cell Signaling Technology (Danvers, MA, United States); anti-HIF-1α antibody was raised in rabbit as previously described^29^.

### Cell culture and transfection

Cells were grown in DMEM or RPMI-1640 media supplemented with 10% fetal bovine serum (FBS) and 1% penicillin-streptomycin. Culture media and FBS were purchased from Invitrogen (Carlsbad, CA, United States) and Sigma–Aldrich (St. Louis, MO, United States), respectively. Penicillin-streptomycin solution was purchased from Biological Industries (Beit HaEmek, Israel). Culture dishes were maintained in a humidified atmosphere under standard conditions of 5% CO_2_ at 37°C. For hypoxic conditions, cells were incubated in hypoxia chamber with 1% O_2_ concentrations. STR DNA cell line profiling was performed in Korean Cell Line Bank (Seoul, South Korea). Mycoplasma contamination was routinely checked when cell shape or growth was altered. Plasmids and siRNAs were transfected with Lipofectamine 3000 and RNAiMAX, respectively, according to the instructions provided by Invitrogen (Carlsbad, CA, United States).

### Plasmids and siRNAs

The human *EPO* enhancer region (5′-GGTACCGGCCCTACGTGCTGTCTCACACAGCCTGTCTGACCTCTCGACCTACCGGCCAGATCT-3′) containing the HIF binding site was cloned into the pGL3-basic vector (Promega, Madison, WI), which was designated as EPO-Luc. A 989-base fragment (−952 to + 37) of the rat *VEGF* promoter (GenBank: U22373.1) containing the HIF binding site was cloned into the pGL3-basic vector, which was designated as pVEGF-Luc. The full-length of *PIN1* cDNA was cloned by RT-PCR using two primers (5′-GAAGATGGCGGACGAGGA-3′ and 5′-TCACTCAGTGCGGAGGATGA-3′), which were inserted into the pcDNA vector (Promega). The 3′-UTR region of the *PIN1* mRNA was cloned by RT-PCR using specific primers (5′-ATCCTCCGCACTGAGTGA-3′ and 5′-GCAGTGGTTCTGGGTTTAATT-3′), which were inserted into the FLAG-tagged pcDNA vector (Promega). The nucleotide sequences of the plasmids were confirmed by standard DNA sequencing. Small interfering RNAs (siRNAs) were designed and purchased from Integrated DNA Technologies (Coralville, IA, United States). The sequences of siRNAs are listed in Supplementary Table 1.

### Western blot analysis

For immunoblot analysis, cell lysates were boiled in sample buffer (125 mM Tris-HCl pH 6.8, 4% SDS, 20% glycerol, 10% 2-mercaptoethanol, 0.004% bromophenol blue) for 5 min at 95°C. Denatured proteins were separated by SDS-PAGE and transferred to Immobilon-P membrane (Millipore, Burlington, MA, United States). Membrane was blocked with 5% skim milk for 1 h at room temperature and probed with primary antibodies overnight at 4°C. The HRP-conjugated secondary antibodies were applied for 1 h at room temperature and bands were visualized by ECL detection reagents (GE Healthcare, Chicago, IL, United States).

### Reporter assays

Reporter assays were performed as previously described^30^. Briefly, cells were co-transfected with β-galactosidase and *EPO* enhancer- or *VEGF* promoter-luciferase plasmids. Luciferase activity was analyzed by Centro LB 960 luminometer (Berthold Technologies, Bad Wildbad, Germany) and β-galactosidase assay was carried out to normalize transfection efficiency.

### RT-qPCR

RT-qPCR was performed as previously described^30^. Briefly, total RNA was isolated from cells with TRIzol reagent (Invitrogen, Carlsbad, CA, United States), and cDNA was synthesized in a reverse transcriptase mixture (Promega, Fitchburg, WI, United States). Real-time PCR was performed using qPCR master mix (Enzynomics, Daejeon, Seoul, South Korea), and fluorescence was analyzed by CFX Connect Real-Time PCR Detection System (Bio-Rad, Hercules, CA, United States). All values were normalized to *GAPDH* expression. The sequences of PCR primers are listed in Supplementary Table 2.

### Semi-quantitative and conventional RT-PCR

Reverse transcription PCR was performed as previously described^31^. Briefly, cDNAs were amplified by PCR and the obtained products were electrophoresed on agarose gels. RNA bands were stained with ethidium bromide and visualized under a UV lamp. The sequences of PCR primers are listed in Supplementary Table 2.

### Chromatin immunoprecipitation (ChIP) assay

ChIP assay was performed as previously described^32^. Briefly, cells were fixed with 1% formaldehyde for 10 min at 37°C. The fixed cells were washed with PBS and centrifuged to collect the crude nuclear fraction. The nuclear pellet was suspended in SDS lysis buffer (50 mM Tris pH 8.1, 10 mM EDTA, 1% SDS) and sonicated. Chromatin complexes were reacted with indicated antibodies overnight at 4°C, and precipitated with protein A agarose/salmon sperm DNA beads for 4 h at 4°C. DNA was isolated by phenol-chloroform-isoamyl alcohol (25:24:1) and precipitated overnight using ethanol and glycogen at −70°C. The extracted DNA was resolved in nuclease-free water and analyzed by qPCR.

### Statistical analysis

Data were analyzed with Microsoft Excel, SigmaPlot or GraphPad Prism software. Experimental results are presented as the means and standard deviations, and significance within the data was determined by Student’s t-test, Mann-Whitney test or log-rank test as indicated. Differences were considered significant at *P* < 0.05.

## Supplementary information

Suppelmentary Information
