# Supplementary material for: PIN1 transcript variant 2 acts as a long non-coding RNA that controls the HIF-1-driven hypoxic response
Source: Sci Rep. 2019 Jul 22;9:10599. doi: 10.1038/s41598-019-47071-1 (PMC6646326; doi:10.1038/s41598-019-47071-1)
Supplement: Supplementary file 1 — Suppelmentary Information [file 41598_2019_47071_MOESM1_ESM.pdf]

PIN1 transcript variant 2 acts as a long non-coding RNA that controls the HIF-1-driven hypoxic response

Yong-Joon Choi[1, 2], Iljin Kim[1, 2, 3, 4], Jae Eun Lee[1, 2, 3], Jong-Wan Park[1, 2, 3, 4]

[1]Department of Biomedical Sciences, Seoul National University College of Medicine, Seoul, Republic of Korea.

[2]Department of Pharmacology, Seoul National University College of Medicine, Seoul, Republic of Korea.

[3]Cancer Research Institute and Ischemic/Hypoxic Disease Institute, Seoul National University College of Medicine, Seoul, Republic of Korea.

[4]BK21-plus Biomedical Science Project, Seoul National University College of Medicine, Seoul, Republic of Korea.

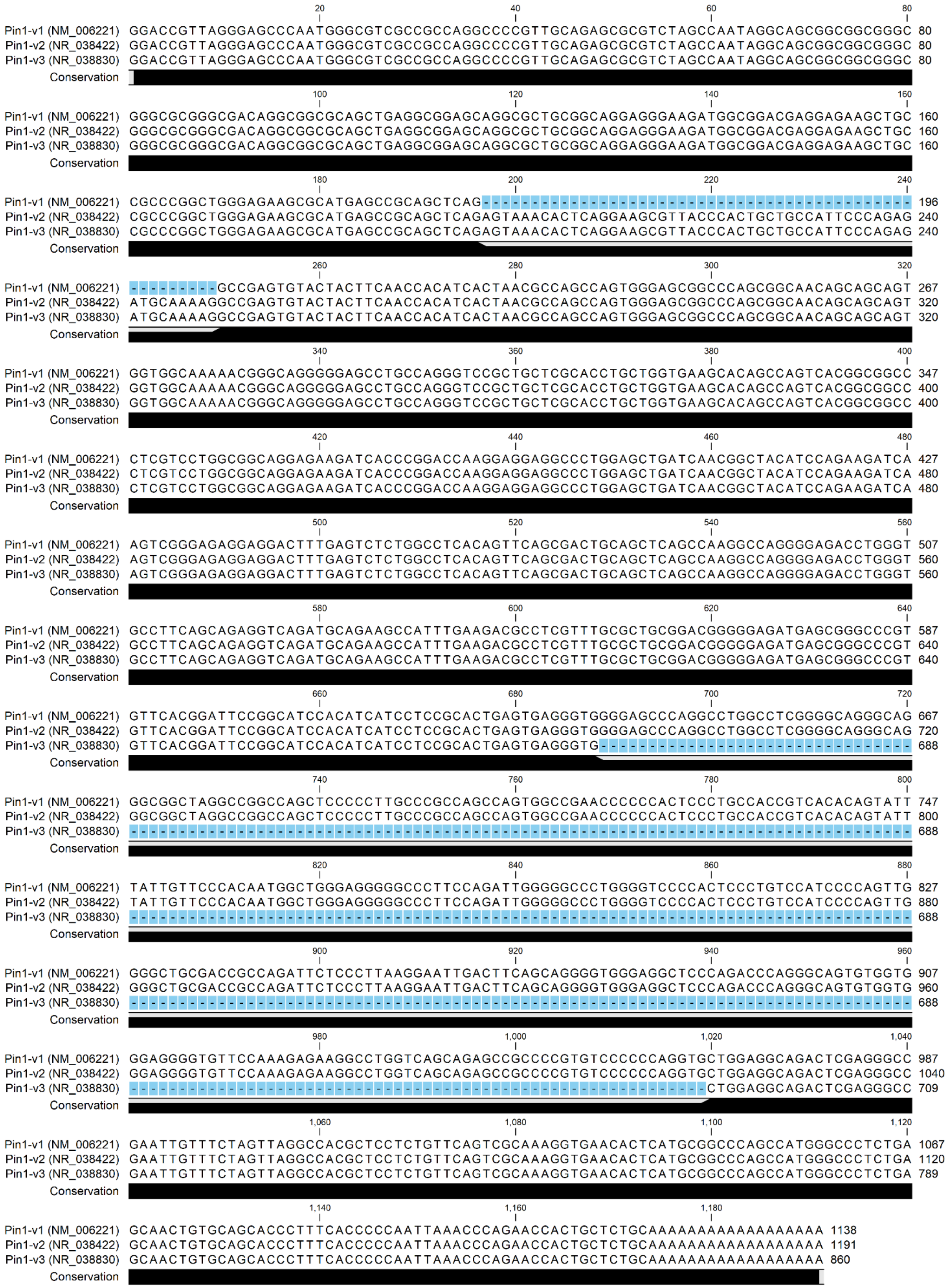

Supplementary Figure S1. Sequence alignment of the *PIN1* transcript variants. Compared to variant 1, variants 2/3 have an additional segment in the 5' region which includes a premature stop codon. In addition, variant 3 lacks a segment in the 3' region.

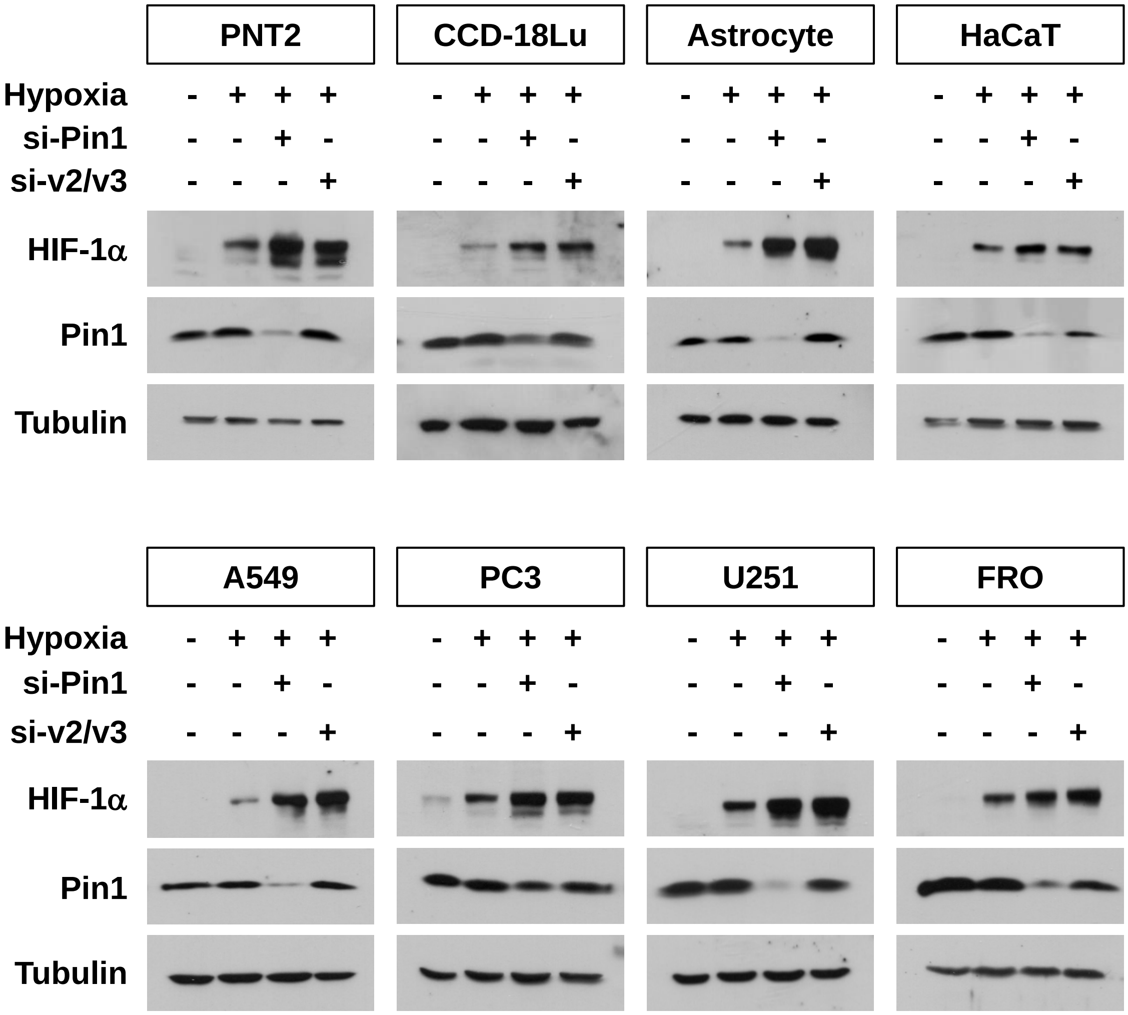

Supplementary Figure S2. Various human cell lines were tested for the effect of si-Pin1 and si-v2/v3 on HIF-1α protein levels. Cells were transfected with the indicated siRNAs and incubated in normoxia (21% O_2) or hypoxia (1% O_2) for 8 h. Cell lysates were analyzed by Western blotting. All experiments were performed three times independently.

(a)

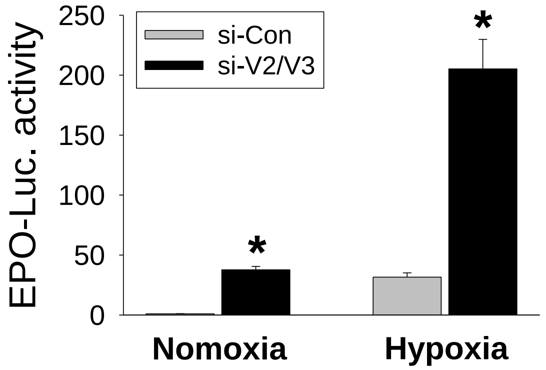

(b)

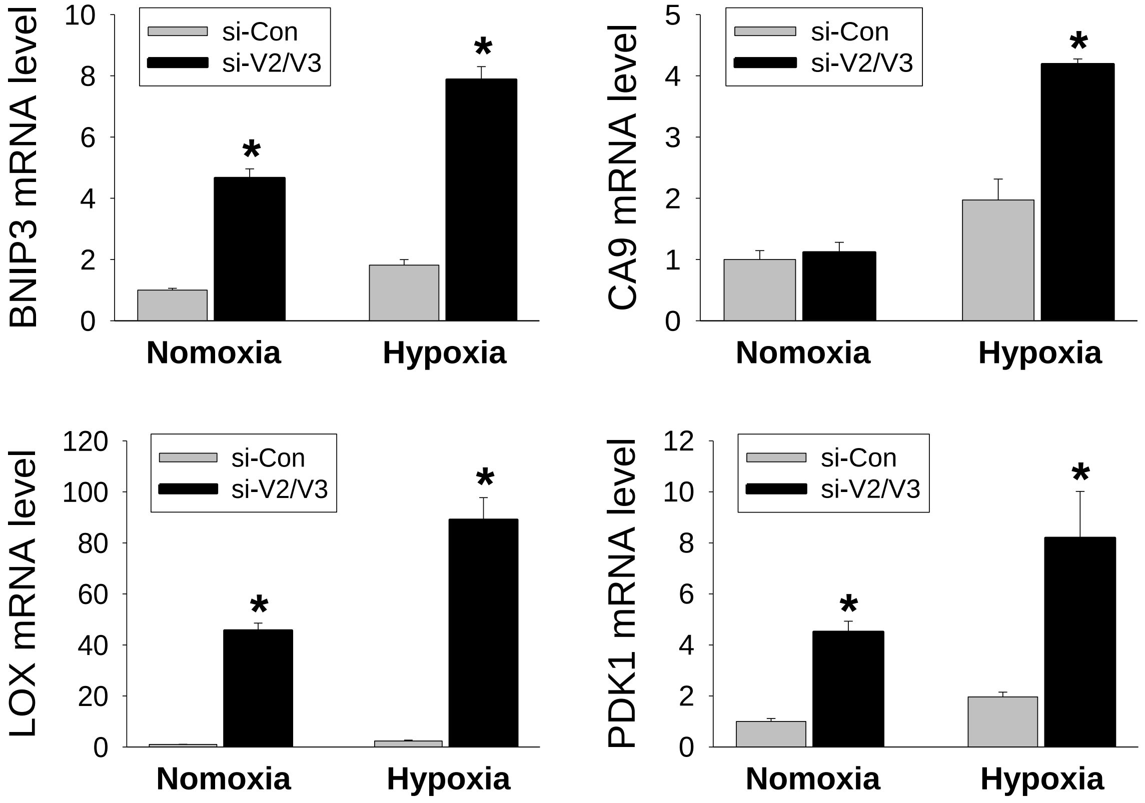

Supplementary Figure S3. (**a**) DU145 cells were transfected with the indicated siRNAs and reporter plasmids and incubated in normoxia (21% O_2) or hypoxia (1% O_2) for 16 h. Luciferase activities were measured and normalized to β-galactosidase activities. Data present mean ± SD (n = 3). * P < 0.05 by Student's t-test. (**b**) Cells were transfected with the indicated siRNAs and incubated in normoxia/hypoxia for 16 h and lysed for RNA extraction. *BNIP3*, *CA9*, *LOX*, and *PDK1* mRNA expression levels were measured by RT-qPCR. Data present mean ± SD (n = 3). * P < 0.05 by Student's t-test.

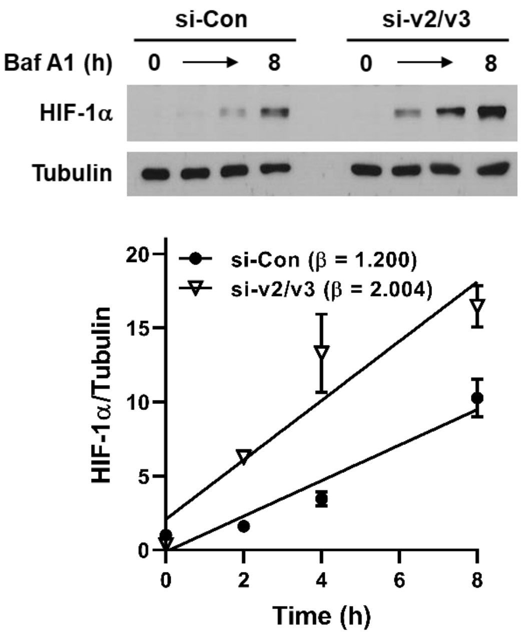

Supplementary Figure S4. HEK293 cells were transfected with the indicated siRNAs and treated with 10 nM Bafilomycin A1. Cells were harvested at various time points (0, 2, 4, 8 h) and analyzed by Western blotting. HIF-1α/tubulin blot intensities (mean ± SD, n = 3) are shown in line graph with calculated slope values (β) at the lower panel.

Supplementary Table 1. List of siRNAs used in this study.

siRNA	Sequence (5'-3')
Control	AUGAACGUGAAUUGCUCAATT
Pin1 #1	UGCCCGUUUUUGCCACCACUGCUGCUG
Pin1 #2	UGGGUUUAAUUGGGGGUGAAAGGGUGC
Pin1 #3	UAGCCGUUGAUCAGCUCCAGGGCCUCC
Pin1 v1/2 #1	AGAUUCUCCCUUAAGGAAUUGACTT
Pin1 v1/2 #2	CCACCGUCACACAGUAUUUAUUGTT
Pin1 v2/v3	UGGGAAUGGCAGCAGUGGGUAACGCUU

Supplementary Table 2. List of primers used in this study.

Target	Sequence (5'-3')
GAPDH	Forward: TGTGGTCATGAGTCCTTCCA<br>Reverse: CGAGATCCCTCCAAAATCAA
BNIP3	Forward: TGTTGCAAGCTCAGAAGTAA<br>Reverse: TTCTGAAAGTTTTCCTTCCA
CA9	Forward: GTGTAGTCAGAGACCCCTCA<br>Reverse: GGAAGAAAACAGTGCCTATG
LOX	Forward: GTAGCCATAGTCACAGGATG<br>Reverse: ACCAAGGGACATCAGATTTC
PDK1	Forward: AACCCTCTAGGGAATACAGC<br>Reverse: CCTTTGAGGAAAATTGACAG
HIF1A	Forward: CCCAGATTCAGGATCAGACA<br>Reverse: ACCATCATGTTCCATTTTTCG
HIF1A pre-mRNA	Forward: GTCTGCGAGAAAACTTTGTAA<br>Reverse: ATGTGTGCATTTTACCTGAGT
HIF1A promoter	Forward: GAACAGAGAGCCCAGCAGAG<br>Reverse: CCTGAGGTGGAGGCGGGTTC
PIN1 v1	Forward: CCGCAGCTCAGGCCGAGT<br>Reverse: CCATTGTGGGAACAATAAAT
PIN1 v2	Forward: ATTCCCAGAGATGCAAAAG<br>Reverse: CCATTGTGGGAACAATAAAT
PIN1 v3	Forward: ATTCCCAGAGATGCAAAAG<br>Reverse: TCCAGCACCCTCACTCAGT
